# Case Report: Durvalumab-Associated Encephalitis in Extensive-Stage Small Cell Lung Carcinoma

**DOI:** 10.3389/fonc.2021.693279

**Published:** 2021-06-24

**Authors:** Yosuke Shionoya, Akito Hattori, Taro Hanada, Michihiro Fujino

**Affiliations:** ^1^ Department of Respiratory Medicine, Tenshi Hospital, Hokkaido, Japan; ^2^ Hanada Hospital, Hokkaido, Japan

**Keywords:** durvalumab, small cell lung carcinoma, extensive stage, encephalitis, immunotherapy

## Abstract

In recent years, the clinical importance of immunotherapy has been demonstrated in the treatment of extensive-stage small-cell lung cancer (ES-SCLC). However, immune checkpoint inhibitors (ICIs) have been shown to cause immune-related adverse events (irAEs), including autoimmune encephalitis. Here, we describe th treatment of a patient with ES-SCLC who developed immune-related encephalitis. A 68-year-old Japanese woman with ES-SCLC treated with carboplatin plus etoposide plus durvalumab 20 days earlier was admitted to our hospital with a high fever and anorexia. Her symptoms gradually worsened over time, and she had a headache daily and showed reduced levels of consciousness. An electroencephalogram showed diffuse slow waves, and there was a slight increase in cell counts and an increase in protein levels in the cerebrospinal fluid. The patient was diagnosed with durvalumab-associated encephalitis. Her symptoms improved immediately after steroid pulse therapy. Following steroid pulse therapy, oral prednisolone (1 mg/kg) was administered, and then, the dose was gradually reduced. Subsequently, treatment with carboplatin plus etoposide without durvalumab was restarted. In conclusion, this study shows the efficacy of steroid therapy in the treatment of durvalumab-induced encephalitis in ES-SCLC.

## Introduction

Globally, almost 20 million new cancer cases were diagnosed and almost 10 million cancer-related deaths were occurred in 2020, with lung cancer being the leading cause of cancer-related deaths (1). Cigarette smoking is the most recognized risk factor for the development of lung cancer; 10%–15% of smokers develop lung cancer, and 20%–30% develop Chronic Obstructive Pulmonary Disease (COPD), which is closely related to lung cancer. Small cell lung cancer (SCLC), which is characterized by a severe clinical course, accounts for 14% of lung cancer cases. It typically presents as a perihilar mass with immediate and extensive lymph node metastases, and it is strongly associated with smoking history and commonly causes paraneoplastic syndrome (2). Several patients with SCLC have an extensive-stage disease at diagnosis, and the prognosis remains poor despite cytotoxic chemotherapy. Recently, immune checkpoint inhibitors (ICIs), such as programmed death ligand 1 (PD-L1) antibody, have demonstrated clinical efficacy in extensive-stage (ES)-SCLC treatment. The CASPIAN trial assessed durvalumab in combination with etoposide with either cisplatin or carboplatin as a first-line therapy for patients with ES-SCLC (3). Various types of immune-related adverse events (irAEs) can affect multiple organs in the body. The probability that patients will develop autoimmune encephalitis when treated with ICIs is low (4), and durvalumab-associated encephalitis has not been reported. Herein, we present a patient with lung cancer who developed autoimmune encephalitis associated with durvalumab.

## Case Presentation

**Figure 1:**
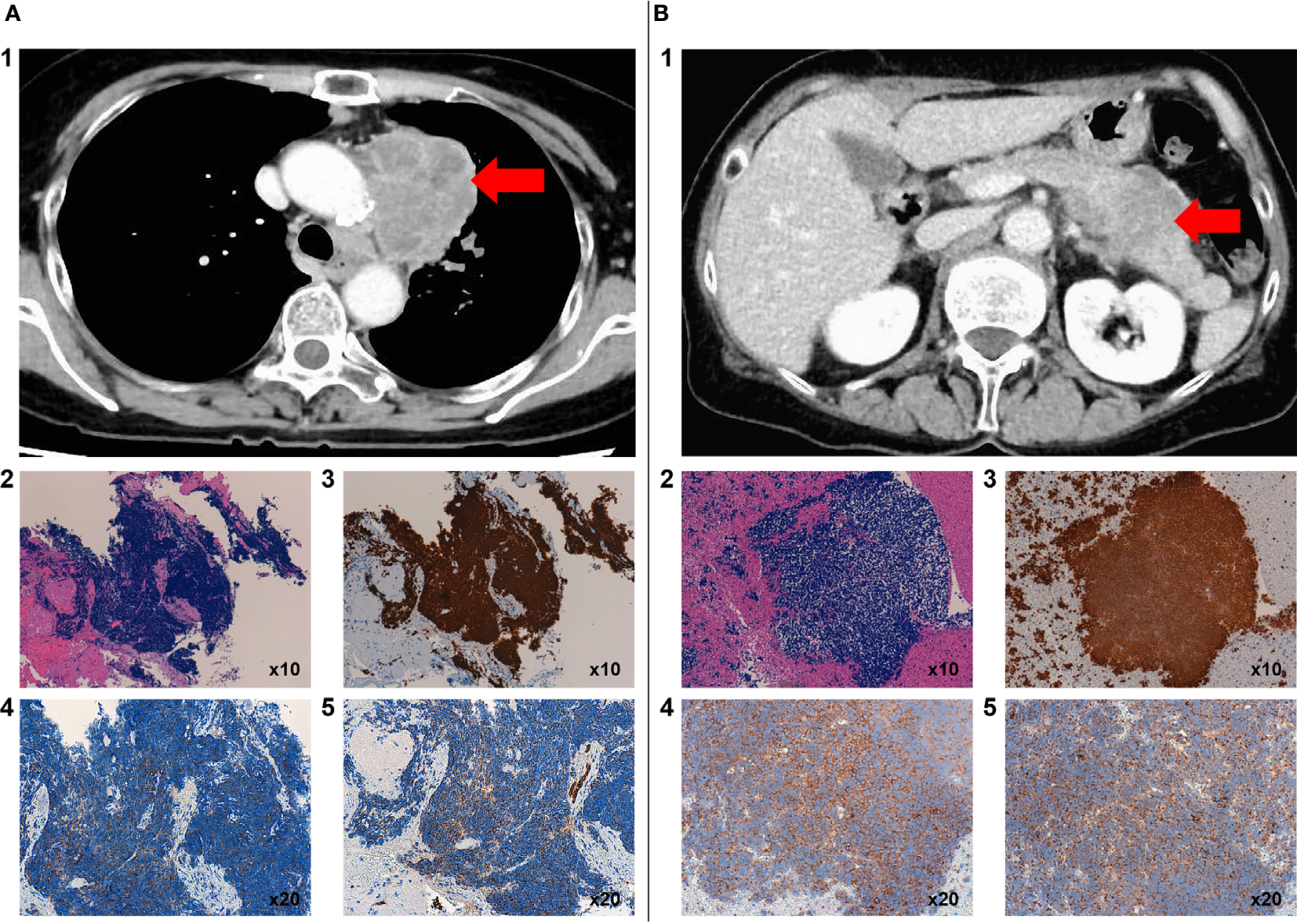
Contrast chest CT scan before chemotherapy showed a primary mass in the upper lobe of the left lung with mediastinal lymph node metastasis **(A1)**. Histology, including immunostaining, of a biopsy specimen of the lung tumor by transbronchial biopsy. Microscopic images of hematoxylin and eosin staining of small cells with a high N/C ratio showed proliferation **(A2)**. TTF-1 **(A3)**, synaptophysin **(A4)**, and AE1/AE3 **(A5)** were positive. Contrast-enhanced abdominal CT scan before chemotherapy showed occupied lesions in the pancreas **(B1)**. Histology, including immunostaining, of a biopsy specimen of the pancreatic tumor by endoscopic ultrasound-guided fine needle aspiration (EUS-FNA) from the stomach. Hematoxylin and eosin staining showed the proliferation of small cells with a high N/C ratio **(B2)**. TTF-1 **(B3)**, synaptophysin **(B4)**, and AE1/AE3 **(B5)** were positive.

A 68-year-old Japanese woman with a 20-pack 48-year smoking history (smoking index: 960) visited a hospital that she regularly visits because of a stomach discomfort. She had a gastric ulcer when she was 48 years old. Her father died of esophageal cancer and her mother died of colorectal cancer. She was suspected to have pancreatic cancer based on the findings of abdominal CT imaging. She was referred to our hospital for a close inspection of the pancreatic tumor. Initially, she was admitted to the Department of Gastroenterology. Contrast chest and abdominal computed tomography (CT) showed lung, pancreatic body, and adrenal gland tumors. Her tumor marker levels were considerably high (Neuron Specific Enolase [NSE]: 209 ng/mL, and Pro-Gastrin-Releasing Peptide [ProGRP]: 3960 pg/mL). The lung tumor was diagnosed as small cell carcinoma *via* a transbronchial biopsy (**Figure 1A**), and the pancreatic tumor was diagnosed as small cell carcinoma *via* an endoscopic ultrasound-guided fine needle aspiration biopsy (**Figure 1B**). Thus, she was diagnosed with primary lung small cell carcinoma with a clinical stage of T4N2M1c (pancreas and adrenal glands). PD-L1 expression was not evaluated in the present study.

**Figure 2:**
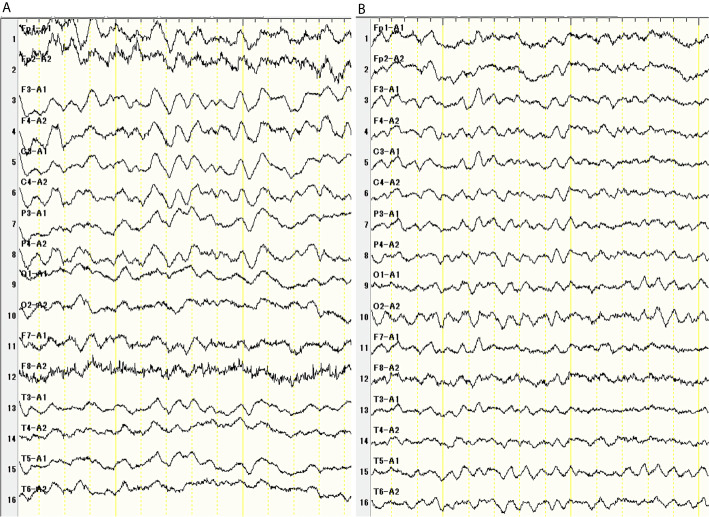
Thirty-one days after chemotherapy, Electroencephalogram showed diffuse slow wave (45 Hz) **(A)**. There were no seizures. EEG improved after steroid pulse therapy **(B)**.

The patient was subsequently transferred to the Department of Respiratory Medicine and treated with carboplatin (AUC = 5 450 mg/body) plus etoposide (115 mg/body) plus durvalumab (1500 mg/body) as the first-line therapy immediately after diagnosis. Seventeen days later, she presented with general weakness and a fever. Twenty days later, she was hospitalized for a persistent fever and general weakness, despite the introduction of amoxicillin/clavulanic acid. The patient had no specific signs or symptoms. On admission, the laboratory examination revealed the following: white blood cell count, 3250 × 10^9^/L; neutrophil, 65.2%; hemoglobin, 12.0 g/dL; platelet count, 31.0 × 104/L; and C-reactive peptide, 5.19 mg/L. The results of the coagulation and hepatic and renal tests were normal. Tumor marker levels were decreased (NSE, 7.5 ng/mL and Pro-GRP, 229 pg/mL). The urine, sputum, and blood cultures were negative. Chest and abdominal CT imaging showed a partial response according to the immune-related Response Evaluation Criteria in Solid Tumors (irRECIST), but showed no inflammation-related findings such as pneumonia, hepatitis, colitis, and pyelonephritis that cause a fever. Heart echo did not reveal vegetation, which cast a doubt on the diagnosis of endocarditis. Transthoracic or transesophageal echocardiography was not performed. Endocrine hormone tests such as T3, T4, TSH, ACTH, and cortisol were normal. Although she was treated with levofloxacin, cefepime, and itraconazole for bacterial or fungal infection after admission, she still had a fever. Twenty-eight days after chemotherapy, she presented with headache and dizziness, without nausea or vomiting. Brain gadolinium contrast magnetic resonance imaging (MRI) showed no abnormalities. Lumbar puncture (LP) showed a mild increase in the cell count (9 cells/mm^3^) with a mixed formula (lymphocytes predominant over neutrophils), elevated protein level (83 mg/dL), and normal glucose level (68 mg/dL). No malignant cells were observed in the cerebrospinal fluid (CSF). CSF culture was negative. Electroencephalography (EEG) showed a slow wave (4–7 Hz) 31 days after chemotherapy (**Figure 2A**). There were no seizures. She presented with disorientation, memory impairment, and eating difficulty. She developed drowsiness and did not respond well to simple questions. She was thus diagnosed with encephalitis. Because of the increased time taken for an accurate diagnosis along with worsening symptoms, the patient and her family became anxious. Constant updates on her circumstances were provided to her and her family to relieve their anxiety. Thirty-two days after chemotherapy, high-dose steroids were introduced (methylprednisolone 1 g/day for 3 days, and then prednisolone 1 mg/kg/day for a week, followed by a gradual decrease) for autoimmune encephalitis. The patient was administered acyclovir (750 mg/day) for 7 days to treat HSV virus encephalitis. The patient recovered completely from the fever and neurological symptoms, and her EEG findings improved (**Figure 2B**). Fifty-six days after the first chemotherapy, she was able to restart carboplatin plus etoposide with 15 mg prednisolone. Durvalumab was stopped. After the second course, she presented with a slight headache, but the CSF test improved: cell count, 5 cells/mm^3^; and protein level, 28 mg/dL). No malignant cells were observed in the CSF. CSF culture was negative. The patient tested negative for HSV virus in the polymerase chain reaction (PCR). As a result, the dose of steroids was increased to 25 mg, followed by a gradual decrease. After four courses, chest and abdominal CT imaging showed a partial response according to irRECIST, and the tumor marker level considerably decreased (NSE 8.9 and 109 pg/mL). During the four courses of carboplatin plus etoposide therapy, the patient’s symptoms such as fever and headache alleviated, and her consciousness level improved; furthermore, there was no recurrence of encephalitis (**Table 1**).

**Table 1:** Characteristics of patients with autoimmune encephalitis associated with anti PD-L1 antibodies, including the present case, four cases from a case report, and cases of Japanese patients who participated in the OAK, IMpower150, and IMpower132 trials based on the appropriate use of atezolizumab from CHUGAI PHARMACEUTICAL CO., LTD.

CSF										
Autoimmune encephalitis cases	Histology	Day	Cell	Cell fraction	Protein	Glucose	MRI abnormal findings	EEG abnormal findings	Therapy	Clinical outcome
Present case Durvalumab	small	17	→	mono	↑	→	–	+	steroid	good
Robert et al. (5) Atezolizumab	adeno	13	↑	mono	↑	↑	+	–	steroid	good
Yamaguchi et al. (6) Atezolizumab	adeno	7	↑	n.r	↑	→	–	n.r	steroid	good
Arakawa et al. (7) Atezolizumab	adeno	13	↑	n.r	↑	→	–	n.r	steroid	good
Laserna et al. (8) Atezolizumab	sq	13	↑	poly	↑	→	+	+	steroid	good
OAK Atezolizumab	NSCLC	14	→	n.r	↑	→	+	n.r	steroid	good
IMpower150 Atezolizumab	NSCLC	16	→	n.r	↑	→	–	n.r	steroid	good
IMpower132 Atezolizumab	NCSLC (non sq)	15	n.r	n.r	n.r	n.r	+	n.r	steroid	good

n.r, not reported; CSF, cerebrospinal fluid; NSCLC, non-small cell lung cancer; MRI, magnetic resonance imaging; small, small cell carcinoma; adeno, adenocarcinoma; sq, squamous cell carcinoma; EEG, electroencephalogram.

## Discussion

Immune checkpoint inhibitors (ICIs) enhance antitumor immunity by blocking negative regulatory components such as programmed cell death protein-1 (PD-1), programmed cell death ligand 1 (PD-L1), and cytotoxic T-lymphocyte associated antigen 4 (CTLA-4). They are highly effective in patients with lung cancer, melanoma, renal cell cancer, urothelial cancer, and other types of tumors. Both atezolizumab and durvalumab are humanized monoclonal antibodies that target programmed death ligand 1 (PD-L1), an inhibitory ligand that negatively regulates T cell activation and proliferation by binding to the PD-1 receptor. The CASPIAN trial assessed the efficacy of durvalumab in combination with etoposide plus either cisplatin or carboplatin, whereas the IMpower133 trial assessed the efficacy of atezolizumab in combination with etoposide plus carboplatin as a first-line ICI with cytotoxic chemotherapy for patients with ES-SCLC.

In both trials, combining ICIs with cytotoxic chemotherapy resulted in significantly longer overall survival and PFS of patients than cytotoxic chemotherapy alone (3, 9). Despite the clinical benefits, ICIs are associated with various adverse effects (10) related to their mechanism of action, which is different from that of cytotoxic chemotherapy.

In addition, ICIs have been associated with various neurological immune-related adverse events such as peripheral neuropathies, Guillain–Barre syndrome, myasthenia gravis, Tolosa–Hunt syndrome, and autoimmune encephalitis. Takahisa et al. reported that among 50,406 ICI-related reports, 7.2% of the reports were pertaining to neurological cases. That is, compared with non-ICI drug use, ICI use is associated with a high risk of neurologic complications (11). Characteristically, ICI-mediated neurological adverse events present early (within approximately 5 weeks) and progress rapidly (12). Thus, clinicians should notice signs immediately and start appropriate treatments as early as possible. It is interesting that human leukocyte antigens (HLAs), especially HLA-B27, are related to atezolizumab-induced encephalitis. HLA-B*27:05 may be a risk factor for CNS-irAEs induced by atezolizumab (13).

Stuby et al. reviewed published case reports of ICI-associated encephalitis and reported their five cases (14). Patients treated with ICIs (ipilimumab, nivolumab, pembrolizumab, atezolizumab, and lambrolizumab) were diagnosed with autoimmune encephalitis. Although durvalumab-associated encephalitis has not yet been reported, this report suggests that all ICIs can cause encephalitis.

In KEYNOTE-189, first-line treatment with pembrolizumab plus pemetrexed-platinum significantly improved the overall survival and progression free survival of patients with NSCLC compared to treatment with placebo plus pemetrexed-platinum. An updated analysis of data from KEYNOTE-189 showed that 2/405 (0.5%) patients who were treated with pembrolizumab plus pemetrexed-platinum developed encephalitis (15).

Although there were no adverse events associated with autoimmune encephalitis in the CASPIAN trial, Impower133 trial (3), or OAK trial (16), a randomized phase III study comparing atezolizumab with docetaxel in patients with previously treated non-small cell lung cancer reported 5/609 (0.82%) cases of encephalitis or meningitis (17). Additionally, in the IMpower130 trial (18), a randomized, phase III study (investigating atezolizumab in combination with carboplatin plus nab-paclitaxel chemotherapy vs. chemotherapy alone as a first-line chemotherapy for metastatic non-squamous non-small-cell lung cancer), 3 of 473 patients (0.63%) developed encephalitis or meningitis. Furthermore, in the Impower 132 trial (19), a randomized, phase III study (investigating atezolizumab plus carboplatin or cisplatin and pemetrexed vs. carboplatin or cisplatin and pemetrexed), 4 of 291 patients (1.37%) developed encephalitis or meningitis. In the Impower 150 study (20), a randomized, phase III study (atezolizumab in combination with carboplatin plus paclitaxel with or without bevacizumab vs. carboplatin plus paclitaxel and bevacizumab), 1 of 393 patients (0.25%) developed meningoencephalitis. These results suggest that atezolizumab- and durvalumab-associated autoimmune encephalitis is a very rare adverse event. Brahmer et al. recommended the management of immune-related adverse events in patients treated with immune checkpoint inhibitor therapy. If encephalitis is suspected, the diagnostic work-up should include neurologic consultation, brain MRI, lumbar puncture, EEG, and blood tests. In the lumbar puncture, the number of cells and the levels of proteins and glucose should be checked, and Gram staining and bacterial pathogen culture should be performed. Furthermore, PCR for herpes simplex virus and other viral PCRs should be carried out depending on suspicion, cytology, oligoclonal bands, autoimmune encephalopathy, and paraneoplastic panels. In EEG, subclinical seizures should be evaluated (10). In the present case, abnormal EEG findings and increased CSF protein levels were the triggers for diagnosis. Because the patient’s symptoms improved immediately after the start of steroid pulse therapy, we did not check for paraneoplastic panels. If the response to steroid therapy is not sufficient, metabolic panel, paired samples of paraneoplastic antibodies, and autoimmune encephalitis antibodies in the serum and CSF should be examined (14).

The severity of neurological adverse events was graded from 1 to 4. In this case, the severity was graded as 3 (severe symptoms); steroid therapy was initiated. In some severe cases of encephalitis induced by ICIs, corticosteroid treatment is followed by immunoglobulin administration, plasma exchange, or rituximab use (10, 14). If the severity of the neurological adverse events is graded as 3–4, ICIs should be stopped per the guidelines. In the present case, the patient was able to restart the carboplatin plus etoposide treatment, and the durvalumab treatment was stopped.

**Figure 3:**
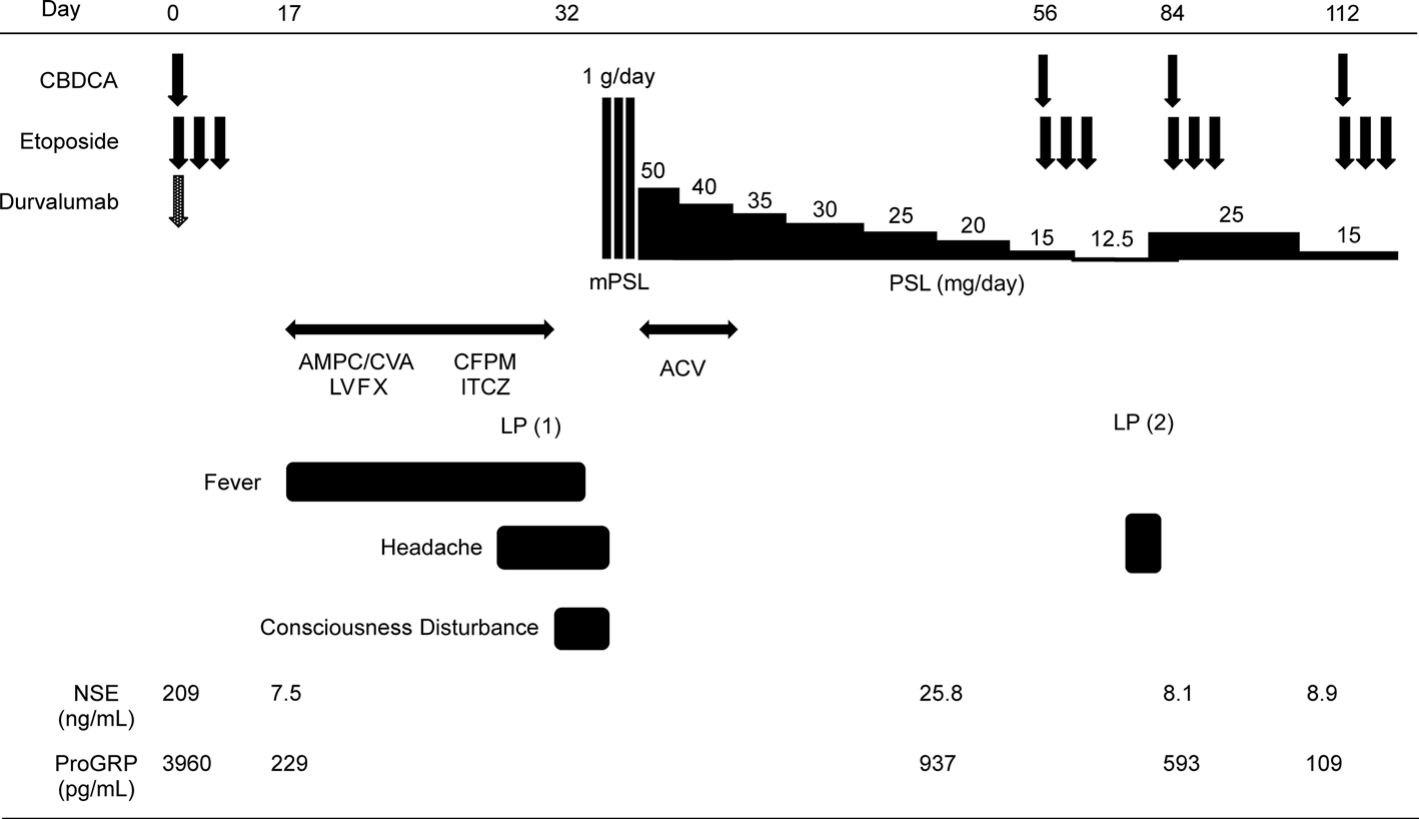
Clinical course of the present case. AMPC/CVA, amoxicillin/clavulanic; LVFX, levofloxacin; CFPM, cefepime; ITCZ, itraconazole; ACV, acyclovir; PSL, prednisolone; mPSL, methylprednisolone.

In eight anti PD-L1 antibody-associated autoimmune encephalitis cases (this case, 4 cases from published case reports (5–8), and 3 cases from a clinical trial), encephalitis was diagnosed 7–17 days after the first course of atezolizumab. Therefore, encephalitis should be considered, especially about 2 weeks after the first treatment course. Even if the MRI and EEG findings are normal, encephalitis cannot be completely excluded. An increase in the protein levels in the CSF is a characteristic of encephalitis. In all cases, the patients recovered with steroid therapy (**Figure 3**). It is important to perform all workups (MRI, LP, and EEG) to diagnose autoimmune encephalitis. The results of LP (cell count, protein, glucose, and Gram stain), MRI, and EEG can be obtained quickly. However, CSF culture, cytology, and HSV PCR are time consuming. Oyanguren et al. reported that herpetic encephalitis and autoimmune encephalitis have similar clinical presentations and radiological findings (21). We cannot completely distinguish autoimmune encephalitis from viral encephalitis even if we perform all workups in patients treated with ICIs who present with rapidly evolving confusion. If encephalitis is suspected, both steroid and antiviral therapies should be started as early as possible, while distinguishing infection, cancerous meningitis, and paraneoplastic syndrome before the conditions of patients worsen.

## Data Availability Statement

The raw data supporting the conclusions of this article will be made available by the authors, without undue reservation.

## Ethics Statement

The studies involving human participants were reviewed and approved by Tenshi Hospital. The patients/participants provided their written informed consent to participate in this study.

## Author Contributions

MF, TH, and YS contributed to the conception and design of the study. AH and YS organized the database. YS wrote the first draft of the manuscript. YS wrote some sections of the manuscript. All authors contributed to the article and approved the submitted version.

## Conflict of Interest

The authors declare that the research was conducted in the absence of any commercial or financial relationships that could be construed as a potential conflict of interest.
